# Shared Decision-Making Tools Implemented in the Electronic Health Record: Scoping Review

**DOI:** 10.2196/59956

**Published:** 2025-02-21

**Authors:** Joni H Pierce, Charlene Weir, Teresa Taft, William Richards II, Mary M McFarland, Kensaku Kawamoto, Guilherme Del Fiol, Jorie M Butler

**Affiliations:** 1 Department of Biomedical Informatics Spencer Fox Eccles School of Medicine University of Utah Salt Lake City, UT United States; 2 Spencer S. Eccles Health Sciences Library University of Utah Salt Lake City, UT United States; 3 Division of Geriatrics Department of Internal Medicine University of Utah Salt Lake City, UT United States; 4 Geriatrics Research, Education, and Clinical Center (GRECC) VA Salt Lake City Healthcare System Salt Lake City United States; 5 Informatics Decision-Enhancement and Analytic Sciences Center (IDEAS) VA Salt Lake City Healthcare System Salt Lake City United States

**Keywords:** shared decision-making, patient engagement, decision support, electronic health records

## Abstract

**Background:**

Patient-centered care promotes the involvement of patients in decision-making related to their health care. The adoption and implementation of shared decision-making (SDM) into routine care are constrained by several obstacles, including technical and time constraints, clinician and patient attitudes and perceptions, and processes that exist outside the standardized clinical workflow.

**Objective:**

We aimed to understand the integration and implementation characteristics of reported SDM interventions integrated into an electronic health record (EHR) system.

**Methods:**

We conducted a scoping review using the methodological framework by Arksey and O’Malley with guidance from the Joanna Briggs Institute. Eligibility criteria included original research and reviews focusing on SDM situations in a real-world clinical setting and EHR integration of SDM tools and processes. We excluded retrospective studies, conference abstracts, simulation studies, user design studies, opinion pieces, and editorials. To identify eligible studies, we searched the following databases on January 11, 2021: MEDLINE, Embase, CINAHL Complete, Cochrane Library including CENTRAL, PsycINFO, Scopus, and Web of Science Core Collection*.* We systematically categorized descriptive data and key findings in a tabular format using predetermined data charting forms. Results were summarized using tables and associated narratives related to the review questions.

**Results:**

Of the 2153 studies, 18 (0.84%) were included in the final review. There was a high degree of variation across studies, including SDM definitions, standardized measures, technical integration, and implementation strategies. SDM tools that targeted established health care processes promoted their use. Integrating SDM templates and tools into an EHR appeared to improve the targeted outcomes of most (17/18, 94%) studies. Most SDM interventions were designed for clinicians. Patient-specific goals and values were included in 56% (10/18) of studies. The 2 most common study outcome measures were SDM-related measures and SDM tool use.

**Conclusions:**

Understanding how to integrate SDM tools directly into a clinician’s workflow within the EHR is a logical approach to promoting SDM into routine clinical practice. This review contributes to the literature by illuminating features of SDM tools that have been integrated into an EHR system. Standardization of SDM tools and processes, including the use of patient decision aids, is needed for consistency across SDM studies. The implementation approaches for SDM applications showed varying levels of planning and effort to promote SDM intervention awareness. Targeting accepted and established clinical processes may enhance the adoption and use of SDM tools. Future studies designed as randomized controlled trials are needed to expand the quality of the evidence base. This includes the study of integration methods into EHR systems as well as implementation methods and strategies deployed to operationalize the uptake of the SDM-integrated tools. Emphasizing patients’ goals and values is another key area for future studies.

## Introduction

### Background

Shared decision-making (SDM) is a model of patient-centered care that encourages patients and clinicians to work together to reach medical decisions by weighing the risks and benefits of various options within the context of the values and goals of the patient [1]. SDM is the process of communication, deliberation, and decision-making between clinicians and their patients [2,3]. During this process, treatment options, including risks and benefits, are discussed and patient preferences are explored to inform decision-making. Previous research has shown that SDM increases patient engagement, knowledge, risk comprehension, and participation in decision-making [4-6]. Furthermore, SDM has been shown to improve patient–health care provider communication, patient satisfaction, compliance, and clinical outcomes [7,8]. Notably, the US Preventive Services Task Force considers SDM an ethical right of all patients, independent of health outcomes [9].

Currently, SDM is not routinely required by large health care payers, such as the Centers for Medicare and Medicaid Services, which is a US federal agency providing health care for patients with low income or older adult patients. Medicare is the largest single payer for health care in the United States [10]. The Centers for Medicare and Medicaid Services is slowly adopting SDM standards for limited and specific procedures, conditions, and devices, including lung cancer screening, atrial fibrillation, and implantable defibrillators [11]. US states such as Washington and Vermont are also beginning to mandate SDM for specific conditions and other states such as Connecticut, Massachusetts, Maine, Minnesota, New Hampshire, and Oklahoma are considering legislation [12].

The Health Information Technology for Economic and Clinical Health Act was adopted in the United States in 2009, enabling the widespread adoption of electronic health record (EHR) systems across the US health care system [13]. Leveraging EHR systems to promote the use and uptake of SDM appears to be a logical approach to operationalizing SDM programs. For example, Kuo et al [7] found several positive effects of integrating SDM tools within an EHR system, which include improved clinical outcomes, positive lifestyle behavior changes, more deliberation with clinicians, and less decisional conflict. Despite the interest in incorporating SDM into routine care, current research studies identify a variety of obstacles that limit SDM adoption. Some of these obstacles include technical integration issues; logistical and workflow challenges; and psychological impediments, such as uncertainty and legacy belief systems, which continue to impede progress [14-16]. Integrating SDM tools and processes into EHR systems is often a complex and difficult problem [17-21].

SDM may involve the use of a decision aid, a type of tool that helps patients consider options for medical decision-making by increasing patient knowledge; illuminating treatment options including risks, benefits, and efficacy; and exploring patient preferences and values [22-24]. The Ottawa Decision Support Framework provides recommendations and guidelines for health care decisions and standardized checklists for the development and evaluation of decision aids based on the International Patient Decision Aid Standards [25]. SDM components found within the International Patient Decision Aid Standards include clarifying patient values, coaching and guiding patients when making decisions, and ensuring literacy levels are addressed [26-28]. Decision aids developed using the Ottawa Decision Support Framework as well as other decision aids outperformed the usual care for performance in a large overview of systematic reviews [25,28]. Patient decision aids can play a key role in facilitating the occurrence of SDM at the point of care as clinicians may use a decision aid to support SDM conversations. Furthermore, decision aids may provide a way to standardize SDM experiences. Notably, decision aid research developed before the widespread adoption of EHRs [29-32]. Decision aids are not synonymous with SDM processes as SDM processes may incorporate the use of a decision aid or may use a conversation between patient and clinician without the use of a decision aid. Decision aids typically contain patient education as part of the decision aid content; however, a clinician may choose to verbally educate a patient without the use of a decision aid. Previous research has shown that decision aids integrated into an EHR system show improvement in patient-centered outcomes, including a reduction in decisional conflict. However, there are significant barriers and complexities to overcome when integrating them into commercial EHR systems [33-35].

Both decision aids and SDM tools still face substantial hurdles to clinician uptake [15,32,36-38]. Gaining the attention of health care providers can be difficult as SDM relies on health care provider discretion, time, and required effort [15,39-41]. Integration into the EHR system is recognized as an important strategy as it enables SDM to be part of clinical workflow [42]. However, integration can also pose challenges because unavoidable health care provider alerts run the risk of high levels of alert fatigue or reactance. SDM tool developers and health care systems must be judicious in their choice of tools to develop and integrate. It is important to understand the characteristics of SDM tools that have been integrated into EHR systems.

### Objectives

A literature review revealed limited studies describing the integration and implementation of SDM tools within an EHR workflow [43-46]. A number of recent systematic reviews have been published on the general topic of SDM [19,33,47-58]. These studies focused on a range of SDM topics, including SDM within specific surgical specialties, behavioral theories underpinning SDM, organizational characteristics to support implementation, validated SDM measurement tools, SDM key components, and quality of SDM. However, at the time of the protocol registration, we were unable to identify a systematic or scoping review that specifically addressed the characteristics of SDM tools that have been integrated into an EHR. Thus, this study aimed to understand the *characteristics of SDM tools* that have been integrated into an EHR system and implemented within a health care system by conducting a scoping review.

## Methods

### Overview

This scoping review followed methods described by Arksey and O’Malley [59], Levac et al [60], and the Joanna Briggs Institute (JBI) [61]. The study protocol is published with the Protocol Registry of Evidence Reviews at the University of Utah. Consensus was achieved on the inclusion and exclusion criteria and primary research question. For transparency and reproducibility, we followed the PRISMA-ScR (Preferred Reporting Items For Systematic Reviews And Meta-Analyses Extension For Scoping Reviews) and PRISMA-S (Preferred Reporting Items For Systematic Reviews And Meta-Analyses Search Extension) reporting guidelines for the scoping review protocol and manuscript [62,63].

### Study Identification

An information specialist developed the search strategies using database-specific subject headings, keywords, and team feedback for the key concepts of SDM, patient engagement, decision support, and EHRs. Example publications were provided to the information specialist for strategy development and term harvesting. The search strategy was evaluated by a librarian coauthor using the Peer Review of Electronic Search Strategies guidelines [64]. EndNote (version 20; Clarivate) was used to manage citations and remove duplicates. Covidence (Veritas Health Innovation) provided a second pass for duplicate removal. Search strategies are provided in Multimedia Appendix 1.

The following databases were used to identify eligible studies between 2009 and 2021: MEDLINE (via Ovid platform), Embase (via Embase platform), CINAHL Complete (via EBSCOhost platform), Cochrane Library (via Wiley platform) including CENTRAL (via Wiley platform), PsycINFO (via EBSCOhost platform), Scopus (via Scopus platform), and Web of Science Core Collection (via Clarivate Analytics). No filters other than date limits were applied. The date limit from 2009 corresponds to the adoption of the Health Information Technology for Economic and Clinical Health Act in the United States [65].

### Study Screening

Covidence software was used for screening. Four researchers independently screened titles and abstracts for inclusion and exclusion criteria and met for discussion. The protocol was modified during this phase to expand inclusion criteria based on discussions among the screeners. For example, after finding a number of design studies and concept papers, we added the requirement that all studies must have SDM tools that have been implemented into a real-world clinical setting. Furthermore, we excluded retrospective studies after identifying studies that focused on a retrospective analysis of clinical notes and activities. Changes to the protocol are listed in Textbox 1. Following the screening process, 303 (10%) of the 3017 articles were identified for full eligibility assessment.

Expanded screening criteria were added to the original inclusion and exclusion criteria identified in the research protocol, as noted in Textbox 1. The additional categories were identified and added as the team recognized additional screening criteria as screening commenced.

**Expanded scoping review inclusion and exclusion screening criteria.**

**Inclusion criteria**

- Original studies, systematic reviews, and meta-analyses
- Decisions made at the point of care
- Electronic health record–integrated shared decision-making tool
- Intervention implemented into a real clinical setting
- Shared decision-making tool outcomes reported
- Studies written in English

**Exclusion criteria**

- Retrospective studies
- Conference abstracts
- Limited measures

### Eligibility Assessment

A team of 5 researchers, including 2 professors with extensive backgrounds in cognitive and sociotechnical informatics (CW and JMB), 1 informatics researcher experienced in sociotechnical design (TT), an informatics student (JHP), and another informatics student (WR II), met on a weekly basis to discuss inclusion and exclusion criteria and extraction goals for research studies. Initially, each researcher was assigned the same 5 studies to assess for eligibility review. Then, the research team discussed and deliberated on whether each study met the established inclusion criteria until the team achieved consensus. A spreadsheet was created with each evaluation component, including basic study characteristics, integration, and implementation methods. Study characteristics included study type, SDM intervention, clinical domain and conditions, measures, outcomes, integration approaches, and implementation processes. The research team discussed the criteria and achieved consensus for spreadsheet components through discussion, debate, and review. The reviewers then evaluated approximately 10 studies per week as a group, using the spreadsheet to determine criteria for analysis against the study aims. After this, the remaining studies were distributed among the team members for a full review of eligibility. We excluded studies that did not report measurable quantitative outcomes, were not integrated into an EHR system, did not involve a shared decision (with or without a decision aid), and were not in person at the point of care. EHR integration for the purpose of this review ranged from scanned PDFs appended to EHR patient records to full technical integration using semantic interoperability methods.

**Figure 1:**
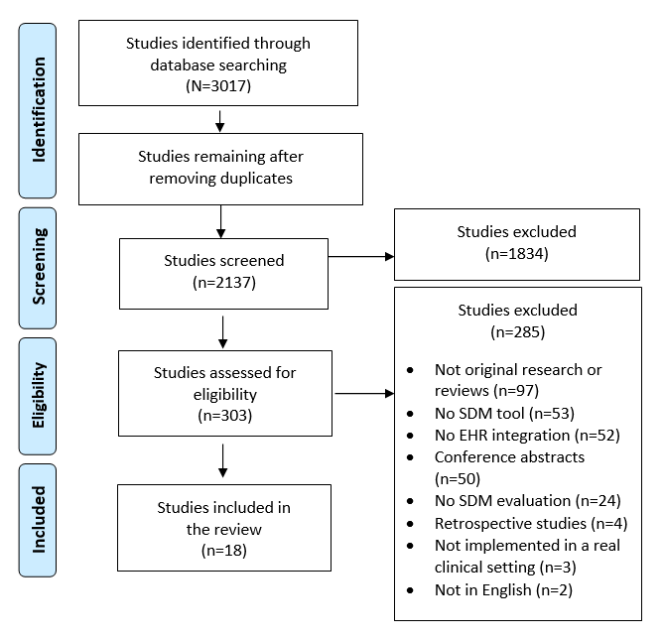
PRISMA-ScR (Preferred Reporting Items for Systematic Reviews and Meta-Analyses Extension for Scoping Reviews) flow diagram. EHR: electronic health record; SDM: shared decision-making.

We identified the studies of tools designed to support SDM that were integrated into the EHR system for evaluation and reported a total of 18 studies, which were included for final review. See PRISMA-ScR Figure 1 and Multimedia Appendix 2.

### Review Process

#### Overview

The research team used the following three-step process to interpret and synthesize the final 18 studies included in the scoping review: (1) data charting, (2) analysis of evidence, and (3) data summary and synthesis. During this process, we performed a detailed review and analysis of the integration methods along with the associated clinical decision support features and then mapped them into a table. Implementation strategies and methods were evaluated and mapped according to the narrative descriptions in the studies.

#### Data Charting

Data charting was performed according to the methods proposed by Arksey and O’Malley [59] and the JBI, as described by Tricco et al [63], Levac et al [60], and Peters et al [66]. The template was piloted by the researchers and adjusted based on discussion, debate, and deliberation. Data items included the following: (1) study design, (2) aims, (3) measures, (4) outcomes, (5) intervention type, (6) clinical domain, (7) clinical processes, (8) clinical conditions, (9) EHR integration methods, (10) implementation strategies, (11) SDM goals, and (12) SDM model and components.

#### Analysis of Evidence

The analysis of the data was then performed to understand and map themes and clusters of notable data according to the updated methodological guidance for scoping reviews [67]. To do this, the research team discussed the definition along with examples of each extraction category. Following this, one researcher (WR II) performed a preliminary extraction on categories that were unambiguous. Next, the remaining research team members were assigned manuscripts to extract the more complex categories. The team met for iterative discussions, deliberation, and decision-making to achieve consensus on the final extraction items.

#### Data Summary and Synthesis

We summarized our findings using narrative synthesis and descriptive statistics to describe the characteristics of SDM tools integrated into the EHR systems [68,69]. For general characteristics, we mapped study types, measures, clinical domains and conditions, and goals of the SDM intervention in a table and calculated frequency counts and percentages [69]. We then categorized each study according to integration methods and features using descriptive content analysis to summarize narrative findings [70]. To do this, we analyzed the various methods used in each of the studies to surface and document SDM tools within the EHR systems. We considered integration to have been achieved so long as the SDM tools surfaced in the clinician’s workflow within the EHR systems. We mapped EHR clinical decision support features for each SDM tool according to risk, patient data display, health care provider notification, documentation, and EHR workflow presentation. Then, we analyzed the implementation approaches across studies. To do this, we looked for methods of creating awareness, training programs, and SDM tool buy-in and promotion through clinician leaders. We were then able to organize our results by general characteristics, integration approaches, and implementation methods.

## Results

### Overview

This scoping review included 18 studies that met the inclusion criteria (Figure 1), including 15 (83%) quantitative and 3 (17%) mixed methods studies. The most commonly used SDM outcome measures are listed in Table 1.

**Table 1:** Shared decision-making (SDM) outcome measures reported in the included studies (N=18).

SDM outcome measures of included studies	Studies, n (%)
SDM measures	7 (39)
Tool use	7 (39)
Clinical outcomes	5 (28)
Patient satisfaction	4 (22)
Documentation	4 (22)
Clinician satisfaction	2 (11)

Of the 18 studies, 7 (39%) measured SDM components, such as patient empowerment, self-efficacy, self-determination, and goal setting. The 7 (39%) studies measuring use rates reported various levels of use; however, predetermined targeted use rate goals were not noted. We found that at least 3 (17%) of the studies achieved a use rate of >50% of the targeted users. In total, 4 (80%) of the 5 studies using clinical outcome measures reported positive improvements based on the SDM intervention [71,72]. Of the 18 studies, 4 (22%) studies that measured patient satisfaction included elements of communication quality, such as helpfulness, respect for the patient’s opinion, understandability, confidence in the health care team, and worry reduction [72-75]. Documentation rates increased for all 4 (22%) studies using documentation as the primary outcome measure [76-79]. A total of 2 (11%) studies that measured clinician satisfaction reported clinician satisfaction at >85% and preference for a systematic approach via a tool at 100% [71,80]. In total, 4 (22%) included studies incorporated patient goals and values in the SDM process [72,78,81,82]. Consistent with previous research, 1 (5%) study reported that obstacles to tool use included a lack of awareness of the tool and low confidence in how to use the tool [83].

### Goals of SDM Intervention

We categorized the included studies by macrolevel functional goals to include (1) care planning and goal setting (10/18, 56% studies), (2) prevention and screening (4/18, 22% studies), and (3) medical management and treatment (4/18, 22% studies; Table 2). The largest percentage of the care planning and goal setting category was end-of-life planning [76,77,81,82], followed by communications [72,84]. In total, 75% (3/4) of the prevention and screening studies focused on the detection of cardiovascular disease [71,74,85], and 75% (3/4) of the medication management and treatment decision-making studies focused on diabetes and related pharmacotherapy [80,83,86].

**Table 2:** Characteristics of the included studies.

Study	Study type	SDM^a^ intervention	Clinical domain and conditions	Measures	Study outcomes
Ballard et al [83], 2017	Quantitative	SCDA^b^ and DMCDA^c^	Primary care Diabetes and hyperlipidemia	Decision aid tool use rates and barriers and facilitators for use	Log file data indicated that 51% of clinicians used the SCDA, and 9% of the clinicians used the DMCDA. Facilitators of decision aid use included clinicians finding them very useful and their impact on treatment decisions. Both facilitators were reported more frequently by clinicians for the SCDA than for the DMCDA (56% vs 30% and 42% vs 22%, respectively). Barriers to use included lack of knowledge of the EMR^d^ link, not finding the decision aids helpful, and time constraints. The use of the tool as intended was low, with many clinicians only discussing decision aid topics that they found relevant.
Bose-Brill et al [76], 2018	Quantitative	A previsit planning tool for patients accessed via a patient portal and EHR^e^-guided point-of-care SDM questions	Primary care ACPf	Documentation of ACP	In total, 19.5% (39/200) of the patients who received previsit planning responded to the framework. The intervention site had an improvement in new ACP documentation rates (*P*<.01) and quality (*P*<.01) among all eligible patients. ACP documentation rates increased by 105% (19/39 to 39/39), and quality improved among all patients who engaged in the previsit planning framework (n=39). Among eligible patients aged between 50 and 60 years at the intervention site, ACP documentation rates increased by 37% (27/96 to 37/96). ACP documentation rates increased by 34% among patients who sent >10 messages per year in the EHR (27/67 to 36/67).
Choi et al [77], 2019	Quantitative	EHR reminder to prompt discussions about unintended cardiac defibrillator shocks and deactivation options for EOL^g^ care	Intensive care Cardiology	Discussion rates and cardiac defibrillator deactivation rates	After the interventions, the rates of discussions regarding the deactivation of ICD^h^ improved from 50% to 93% in patients in comfort care and from 32% to 70% in DNR^i^ patients. The rates of deactivated ICDs improved from 45% to 73% in patients in comfort care patients and from 29% to 40% in DNR patients.
Chunchu et al [78], 2012	Mixed methods	PCCP^j^ tool to promote self-management planning	Family medicine	Self-management plan documented in the EHR Thematic analysis	More frequent documentation was noted in 8 problem-solving elements within the EHR, and the number of concerns for a visit was twice as high as in the control group.
Coylewright et al [85], 2020	Quantitative	iPDAs^k^	Multiple specialties, stroke prevention in atrial fibrillation, fracture prevention in osteoarthritis, breast cancer screening, and lung cancer screening	Clinicians’ use of SDM tools and their perceptions of the tool’s impact on the quality of discussion and time demands	For the 8-year analysis period, 1209 clinicians used iPDAs, with 57,116 unique patients in 81,728 visits. Of the clinicians, 76% were physicians, 16% were APPs^l^, and 8% were other clinicians. There were 2607 unique patient-clinician uses in 2010; 7966 in 2014; and 24,384 in 2017. There was an average increase of 151 new clinicians per year (range 99-302). On average, 82 new clinicians used the iPDAs with at least 1 patient each year (range 56-108), with 657 clinicians for the 8-year period. In total, 54.3% of the clinicians used iPDAs with at least 5 patients.
Crosby and Gutierrez [81], 2019	Quantitative	ACP tool with risk identification for patients with late-stage heart failure	Ambulatory care and congestive heart failure	Use of EOL template and clinician survey	In total, 35% use of templates was reported over 3 months, 100% of templates were documented in EHR, 100% of clinic staff reported inconsistent processes for ACP discussion, and 100% of clinic staff preferred a systematic approach to initiating ACP processes.
Denig et al [80], 2014	Quantitative	Diabetes decision aid	Primary care Type 2 diabetes	Patient empowerment, diabetes management, and lipid-regulating prescription changes	Patient empowerment score increases were 0.1 on a 5-point scale, and drug treatment was intensified in 25% of intervention participants with increased cholesterol levels.
Fiks et al [73], 2015	Quantitative	My Asthma clinical decision support tool	Primary care Asthma	My Asthma tool use and number of missed days of school or work	In total, 57% of the parents in the intervention group used My Asthma during at least 5 of the 6 study months. Parents of children with moderate to severe persistent asthma used the portal more often than others; 92% were satisfied with My Asthma. Parents reported that portal use improved their communication with the office.
Fossa et al [84], 2018	Quantitative	OpenNotes patient access for SDM	Primary care General practice	A 3-item Collaborate scale, which measures a patient’s experience with SDM	A total of 6913 patients responded (28% response rate). Patients reading ≥4 clinical notes in the past 12 months were 17% more likely to have top Collaborate scores when compared to those who had not read a note in the previous 12 months (response rate: 1.17, 95% CI 1.04-1.32).
Fried et al [87], 2017	Quantitative	TRIM^m^	Primary care Pharmacotherapy	Patient involvement in care, patient-clinician communication, and changes in medications	In total, 29.7% of TRIM participants and 15.6% of control participants provided the highest PACIC^n^ ratings; this difference was not significant. TRIM improved communication about medications and the accuracy of documentation.
Huang et al [75], 2020	Quantitative	TEA^o^ tool with an annual assessment of key transition skills and setting of transition skill goals	Pediatrics Gastroenterology	Health care providers’ TEA tool utility ratings and recommendations to patients	All patients (N=53) performed the transition skills self-assessment and practicum and set transition goals with their health care provider. On a scale of 0 (not helpful at all) to 10 (very helpful), patients reported median (IQR) utility scores of 8 (7-10) for the transition readiness assessment, 9 (7-10) for transition resources provided, and 9 (7-10) for the medical history summary. Most (91%) patients would recommend TEA to other patients.
Jouni et al [74], 2017	Quantitative	CHD^p^ genetic risk tool, decision aid for CHD risk estimates, and facilitation of SDM for statin use	Biobank cardiology data Genetic heart diseases	Satisfaction with clinical visits, quality of discussion or participation in decision-making, and physician visit satisfaction	There were no statistically significant differences between the 2 groups in the SDM score, satisfaction with the clinical encounter, and perception of the quality of the discussion or participation in decision-making and physician visit satisfaction scores.
Lafata et al [79], 2014	Quantitative	5A^q^ framework–guided discussion during primary care visits for CRC^r^	Primary care CRC screening	Documented CRC screening rates	In total, 93% of patients received a recommendation for screening (advise), and 53% were screened in the following year. The likelihood of screening increased as the number of 5A steps increased: compared to patients whose visit contained none of the 5A steps, those whose visit contained 1 to 2 steps (OR^s^ 2.96, 95% CI 1.16-7.53), and ≥3 steps (OR 4.98, 95% CI 1.84-13.44) were significantly more likely to use screening.
Litzelman et al [82], 2017	Mixed methods	GW^t^ cards and discussions to identify priorities for advanced care planning	Geriatrics EOL care	ACP conversations completed	A total of 86 patients’ data indicated that they had engaged in a preferences-for-care process using GW R cards. The top 3 card choices by patients were attending to spirituality and religious concerns, preparing for EOL, and maintaining personal wholeness.
Plößnig et al [86], 2015	Quantitative	EMPOWER^u^ SMP^v^ tool to foster self-management of diabetes	Internal medicine Type 2 diabetes	Tool use and empowerment measures: (1) meaningfulness, (2) self-efficacy, (3) impact, and (4) self-determination	Patients used functions for setting goals (65%) or planning activities (71%). More than half of the patients reported using the charts, the calendar, and the journals, while review (29%) and information material (24%) were of little interest. Initial testing showed an overall high level of empowerment. Improvements in self-efficacy, impact, and self-determination were observable in the German users and meaningful in the Turkish users.
Schafer et al [88], 2016	Mixed methods	Pharmacist-led SDM template for medication decisions	Outpatient Pharmacy Mental health	Feasibility of template, type, and number of DTPsw	On average, 2 DTPs were identified per consultation and were most commonly related to appropriate compliance (30%), appropriate indication (26%), medication effectiveness (23%), and safety (21%).
Sona et al [72], 2020	Quantitative	Family communication tools, including education, decision-making, surrogate decision makers, and advanced directives	Critical care	Communication quality, helpfulness of family care conferences, confidence in health care team, impact on patient care, and reduced worry about family members	Excellent communication rating was reported by patients at 69% for RNs^x^ and 54% for physicians; 43.5% of patients or families reported liking family care conferences, 52.2% thought family care conferences were helpful, 62.5% had confidence in health care team, 62.5% reported that family communication impacted care of the patient, and 62.5% reported that it reduced worry about family members.
Sperl-Hillen et al [71], 2018	Quantitative	CV Wizard, an SDM tool to reduce cardiovascular risk	Primary care Cardiovascular disease	10-year cardiovascular risk trajectory and clinician satisfaction rates	Health care provider satisfaction was 85% to 98% for various aspects of the intervention, 10-year cardiovascular risk reduction at 12 months was –2.24%, and use rates were 71% to 77% over the intervention.

^a^SDM: shared decision-making.

^b^SCDA: statin choice decision aid.

^c^DMCDA: diabetes medication choice decision aid.

^d^EMR: electronic medical record.

^e^EHR: electronic health record.

^f^ACP: advanced care planning.

^g^EOL: end of life.

^h^ICD: implantable cardioverter defibrillator.

^i^DNR: do not resuscitate.

^j^PCCP: patient-centered care plan.

^k^iPDA: integrated patient decision aid.

^l^APP: advanced practice provider.

^m^TRIM: tool to reduce inappropriate medication.

^n^PACIC: patient assessment of chronic illness care.

^o^TEA: transition electronic health record activity.

^p^CHD: coronary heart disease.

^q^5A: assess, advise, agree, assist, and arrange.

^r^CRC: colorectal cancer.

^s^OR: odds ratio.

^t^GW: Go Wish.

^u^EMPOWER: Support of Patient Empowerment by an intelligent self-management pathway for patients.

^v^SMP: self-management pathway.

^w^DTP: drug therapy problem.

^x^RN: registered nurse.

Tool objectives were generally a direct function of the use of the SDM tool; for example, an end-of-life planning template may have a stated objective to increase documentation rates for communications. Patient satisfaction–related outcomes, such as decision satisfaction because of a shared conversation, were not noted in 11 (61%) of the 18 studies [72,73,75,80-84,86-88].

### Promoting SDM Awareness and Adoption

#### EHR and Workflow Integration

**Figure 2:**
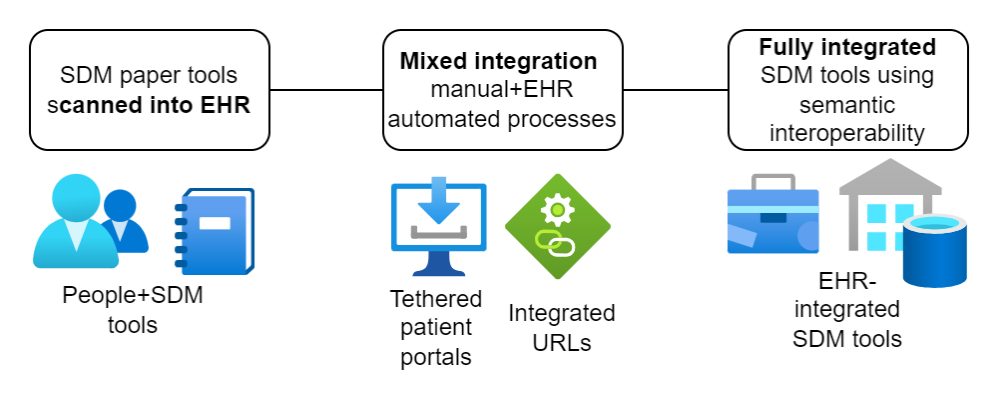
Electronic health record (EHR) integration methods described in the included studies. SDM: shared decision-making.

Our study found that workflow integration approaches were highly variable, ranging from scanning paper-based tools into the EHR system to implementing SDM tools with full semantically interoperable data elements surfacing within patient portals and in the clinicians’ EHR workflow (Figure 2 and Table 3).

**Table 3:** Shared decision-making (SDM) interventions and goals, electronic health record (EHR) integration methods, and implementation approaches (N=18).

Functional goals and studies		Clinical processes	SDM interventions	SDM goals	EHR integration method and implementation approach
**Care planning and goal setting (n=10, 56%)**					
	Bose-Brill et al [76], 2018	ACP^a^	A previsit planning tool for patients accessed via a patient portal and EHR-guided point-of-care SDM questions	To increase the rate of ACP documentation	An EHR-tethered patient portal presents ACP questions before a visit. The algorithm within the EHR guides the communication between clinician and patient. Physicians, nurses, and other clinical staff at the intervention clinic were collectively involved in developing this ACP previsit planning algorithm that was rolled out practice wide over a 3-month period.
	Choi et al [77], 2019	End-of-life planning	EHR clinician alerts and reminders to prompt discussions regarding unintended cardiac defibrillator shocks and deactivation options during end-of-life care	ICD^b^ deactivation discussion	EHR alerts and decision support tools were integrated into the comfort care order set and DNRc document. A 10-min training with pre- and postknowledge assessment, posttraining surveys with follow-up education, and special training sessions for various specialties were provided, as needed.
	Chunchu et al [78], 2012	PCCP^d^	PCCP tool to promote self-management planning	To increase collaborative self-management planning	PCCP was developed in EHR with prompts. Clinician training with video demonstration was provided.
	Crosby and Gutierrez [81], 2019	ACP	ACP tool with risk identification for patients with late-stage heart failure	Risk stratification, life expectancy of <3 years, conversations about preferences, and scanning into EHR	ACP tool was scanned into the EHR. Readiness to change assessment tool was administered to all clinic staff and internal stakeholders. Education was provided about the ACP tool and project goals. Key stakeholders were involved in tool development and project implementation, workflow assessment, and process development.
	Fossa et al [84], 2018	Communication	OpenNotes patient access for SDM	Collaborative decision-making	OpenNotes access was provided via the patient portal.
	Fried et al [87], 2017	Medication reconciliation	TRIM^e^	To facilitate clinician and patient medication communications	TRIM was fully embedded into the EHR and extracted patient data from the EHR for medication reconciliation.
	Huang et al [75], 2020	Transition readiness	TEA^f^ tool with annual assessment of key transition skills and setting of transition skill goals	Transition readiness decision-making	The TEA tool was developed fully in the EHR. Pilot-testing of the TEA tool was performed at an independent clinic.
	Litzelman et al [82], 2017	End-of-life planning or ACP	Go Wish cards and discussions to identify priorities for ACP	ACP conversations	Go Wish cards+ACP discussion took place with EHR data entry of goals and values. Training was provided on ACP and Go Wish R cards through workshops, simulation sessions, case conferences, and guidance from the CDSg tool.
	Schafer et al [88], 2016	Medication reconciliation	Pharmacist-led SDM template for medication decisions	To promote patient and pharmacist SDM with the use of the Ottawa Personal Decision Guide	Pharmacy SDM conversation template was integrated into EHR and printed for the patient.
	Sona et al [72], 2020	Communication	Family communication tools, including education, decision-making, surrogate decision makers, and advanced directives	To increase the rates of critical care family communications	The communication bundle document template was developed in the EHR system.
**Prevention and screening (n=4, 22%)**					
	Coylewright et al [85], 2020	Risk assessment and prevention	iPDAs^h^	To promote clinician use of iPDAs with patients for SDM	A suite of decision aids was integrated into the EHR with use tracking. A clinician adopter survey was conducted.
	Jouni et al [74], 2017	Risk assessment and prevention	CHD^i^ genetic risk tool; decision aid for CHD risk estimates and facilitation of SDM for statin use	Visual comprehension of CHD risk and associated risk reduction with statins	GDMSj, a web-based tool, was integrated using a web viewer into the EHR system.
	Lafata et al [79], 2014	Risk assessment and prevention	5A^k^ framework–guided discussion during primary care office visits for CRC^l^ screening	Use of 5As during clinician-patient discussions to support SDM	Previsit patient survey and clinic visit discussion were guided by EHR prompts.
	Sperl-Hillen et al [71], 2018	Risk assessment and prevention	CV Wizard: a point-of-care CDS tool to facilitate discussion and SDM to reduce cardiovascular risk	Treatment options discussion per printed document	A CDS tool per an EHR-integrated web service
**Medical management and treatment (n=4, 22%)**					
	Ballard et al [83], 2017	Pharmacotherapy	SCDA^m^ and DMCDA^n^	SDM for cholesterol reduction treatment options and SDM for diabetes treatment options	EHR embedded links were sent to decision aids with use tracking.
	Denig et al [80], 2014	Pharmacotherapy	Diabetes decision aid	Treatment options discussion	Decision aid software was integrated into the EHR and populated with individual patient data. Pre- and postclinic visit surveys and health care provider interviews were conducted.
	Fiks et al [73], 2015	Treatment compliance	My Asthma CDS tool	Patient-reported data collection via the My Asthma tool on the patient portal	My Asthma was fully integrated into the EHR for both patients and health care providers. All 3 primary care practices received in-person training on the portal from a physician leader.
	Plößnig et al [86], 2015	Pharmacotherapy	EMPOWER^o^ SMP^p^ tool to foster self-management of diabetes	Self-management of diabetes	EMPOWER SMP was integrated using ISOq 13606 and openEHR information models to support full semantic interoperability.

^a^ACP: advanced care planning.

^b^ICD: implantable cardioverter defibrillator.

^c^DNR: do not resuscitate.

^d^PCCP: patient-centered care plan.

^e^TRIM: tool to reduce inappropriate medication.

^f^TEA: transition electronic health record activity.

^g^CDS: clinical decision support.

^h^iPDA: integrated patient decision aid.

^i^CHD: coronary heart disease.

^j^GDMS: generic disease management system.

^k^5A: assess, advise, agree, assist, and arrange.

^l^CRC: colorectal cancer.

^m^SCDA: statin choice decision aid.

^n^DMCDA: diabetes medication choice decision aid.

^o^EMPOWER: Support of Patient Empowerment by an intelligent self-management pathway for patients.

^p^SMP: self-management pathway.

^q^ISO: International Organization for Standardization.

We found that 8 (44%) of the 18 included studies described implementation strategies in sufficient detail to define the approach to operationalizing the SDM processes [71,73,75,77,78,80,82,85]. In 2 (11%) studies, patient portals were leveraged as part of the SDM process [73,84]. One patient portal was described as a tethered patient portal, which referred to SDM processes that were partially embedded into the clinicians’ EHR view. This was accomplished by collecting data and then saving them into the patient’s record so that a clinician could view the data from their workflow screen [76]. One intervention simply scanned paper documents and uploaded them into the patient record or other sections predefined within the EHR [81].

Some (5/18, 28%) of the studies noted hybrid approaches to EHR integration, such as a combination of manual data collection with EHR documentation of an SDM process (Table 3). Of the 18 studies, 2 (11%) noted the integration of a URL into the EHR that launched a third-party web-based SDM tool [83,85]. Other (12/18, 67%) studies included health care provider alerts that presented patient risk factors to facilitate discussion and decision-making. Health care provider alerts may also help capture documentation of the SDM interaction [74,78].

Determining when to engage a patient in SDM before a clinical encounter is another element of SDM intervention planning. In 1 (6%) study, the engagement interval with patients was extended to previsit preparatory activities [76]. This included SDM processes that initially engaged the patient through preparatory SDM tools or surveys that were delivered via email, patient portals, or web links or delivered in the waiting room immediately before the clinical encounter [73,74,76,79,83-85,87]. Other SDM interventions were designed to be used during the clinical encounter, including EHR-based tools, patient record sharing, communication and conversation guides, patient health plans, and educational printouts for patients [71,72,75,77,78,80-82,86,88]. None of the included studies investigated postclinical encounter SDM tools for ongoing decision affirmation and goal alignment.

#### Implementation Strategies

A total of 9 (50%) of the 18 included studies described implementation strategies in sufficient detail to define the approach to operationalizing the SDM processes [71,73,75,77,78,80-82,85]. Methods to make clinicians and patients aware of the tools included email notifications and reminders, links in the EHR to an external third-party SDM tool, paper-based posters displayed in common workspaces, educational presentations, and training (Table 3). Trainings were often conducted in person, but some used prerecorded video. Brief trainings focused on how to use the tool; however, longer trainings included skills for engaging in SDM and sometimes involved role-playing these behaviors. In one case, the required implementation workflow caused users to question the “long-term feasibility” of the SDM intervention due to the additional time and resources required to complete the SDM processes, specifically in the case of advanced care and end-of-life planning for patients with heart failure [81].

Clinical adoption was supported by collaboration with physician and nursing leaders for integrating the tools into the clinical workflow, engaging clinicians who initially engaged patients when starting the SDM process, providing feedback at the health care provider or clinic level, and sponsoring lotteries for clinic leadership when tool use achieved a specified level.

Other implementation strategies used to support patient engagement with SDM tools included email reminders to complete surveys, the provision of kiosks in the waiting room to allow patients to access the patient portal, and the provision of paper-based information for patients to read while waiting in the examination room.

We found that there was variance across the targeted users for the SDM tool interaction. In several (7/18, 39%) studies, both patients and clinicians had roles to play within the SDM processes [73,75,76,78,82,84,86]. In most cases, the tool was targeted toward the clinician to activate the process and conduct conversations with the patient [71,72,74,77,79-81,83,85,87,88].

#### EHR Clinical Decision Support Features

The SDM interventions identified in this review contained several clinical decision support features. Table 4 describes each of these approaches, along with indicating whether the component was delivered manually or automatically within the EHR system. These features include the following: (1) patient risk alerts, which use patient data to compute personalized risk; (2) patient data display through the retrieval of relevant patient information into an informative display; (3) health care provider notification indicating whether a health care provider has to seek out the SDM tool or whether it surfaces within the EHR automatically based on a set of criteria; (4) presented in the EHR, allowing clinicians to access an SDM tool within the EHR (through several different technical approaches), as opposed to an application that is external to the EHR system; and (5) documentation support, including manual, autogenerated, or template-based clinician notes within an EHR to document that an SDM event occurred. One study used an emoji in the EHR to indicate that the SDM conversation had occurred [72].

**Table 4:** Electronic health record (EHR) clinical decision support features by study (N=18).

Studies	Patient risk calculator+alert		Patient data display		Health care provider notification		Presented in EHR		Documentation		
	Manual (n=6, 33%)	EHR (n=11, 61%)	Manual (n=7, 39%)	EHR (n=13, 72%)	Manual (n=6, 33%)	EHR alert (n=12, 67%)	No (n=3, 17%)	Yes (n=14, 78%)	Manual (n=10, 56%)	Automatic (n=5, 28%)	Template (n=4, 22%)
Ballard et al [83], 2017		✓		✓		✓		✓	✓		
Bose-Brill et al [76], 2018	✓		✓		✓			✓	✓		
Choi et al [77], 2019		✓		✓		✓		✓	✓		
Chunchu et al [78], 2012	✓		✓		✓			✓	✓		✓
Coylewright et al [85], 2020		✓		✓		✓		✓		✓	
Crosby and Gutierrez [81], 2019		✓	✓		✓		✓		✓		✓
Denig et al [80], 2014	✓			✓		✓		✓	✓		
Fiks et al [73], 2015		✓		✓		✓		✓	✓		
Fossa et al [84], 2018				✓							
Fried et al [87], 2017	✓		✓	✓	✓	✓	✓				
Huang et al [75], 2020		✓	✓	✓		✓		✓		✓	
Jouni et al [74], 2017		✓		✓		✓		✓		✓	
Lafata et al [79], 2014		✓		✓		✓		✓			
Litzelman et al [82], 2017	✓		✓		✓			✓	✓		
Plößnig et al [86], 2015		✓		✓		✓		✓	✓		
Schafer et al [88], 2016		✓		✓		✓		✓	✓		✓
Sona et al [72], 2020	✓		✓		✓		✓		✓		✓
Sperl-Hillen et al [71], 2018		✓		✓		✓		✓		✓	

## Discussion

### Principal Findings

This review summarizes the findings of 18 studies that investigated approaches to the integration and implementation of SDM tools within EHR systems. Prominent aspects found across studies included functional SDM goals, such as communication, documentation of communication and discussions, and SDM template use. We identified only 4 (22%) randomized controlled trials (RCTs), which may be a reflection of the operational nature of the study measures. In 10 (56%) studies, clinicians were the primary targeted users, whereas 4 (22%) studies targeted patients and 5 (28%) targeted both. Integrating SDM interventions into a clinician’s workflow within an EHR system appears to support improved SDM uptake [60,89-91]. Studies reported multiple EHR and workflow integration approaches, which may be an indication that a dynamic model that is scalable could help guide the adoption of SDM tools and processes across different environments. Future research to understand the utility of adjusting SDM model depth and complexity based on the magnitude of the decision, the complexity of the clinical situation, and the patient preferences is warranted.

### Goals of SDM Tools

Most (10/18, 56%) of the included studies focused on SDM tools that were functionally designed to support care planning and goal setting. We also noted 1 (6%) study that used the concept of open medical notes as a precursor to shared care conversations.

Most (4/18, 22%) prevention and screening studies targeted by SDM focused on common conditions, such as the detection of heart disease, colorectal cancer, and other common conditions. Future opportunities for EHR-integrated SDM screening tools include the assessment of social determinants of health for the purpose of supporting individuals with social needs. Furthermore, mental health SDM screening tools offer an important area for prevention and screening to detect anxiety, depression, loneliness, addiction, and suicide risk, as a few examples.

### Promoting SDM Awareness and Adoption

Although studies generally lacked sufficient technical detail on the actual integration method into each EHR system, we found considerable variation in how SDM tools are implemented within EHR workflows. The implementation approaches for SDM applications showed varying levels of planning and efforts to promote SDM intervention awareness. For example, 33% (6/18) of the studies did not describe implementation activities for clinicians, whereas other (6/18, 33%) studies described more detailed implementation plans that included training, demonstrations, knowledge queries, and incentives [71,73,77,78,81,82].

In total, 22% (4/18) of the studies included in the review suggested that integrating an SDM intervention into a clinician’s EHR workflow may support increased awareness of the intervention [60,72,89,90]. This represents an important step toward supporting clinicians in the adoption and use of SDM tools and processes. Creating awareness, interest, and commitment to SDM tool use is often overlooked and may contribute to waning adoption for otherwise well-designed SDM tools, potentially leading to adoption failure. The implementation plan is a critical component to moving people and the organizational culture toward the adoption of SDM tools. These steps also provide a forum for direct feedback should clinicians lack confidence in the accuracy or efficacy of an SDM tool, as was noted by Ballard et al [83].

This review found that established processes and required patient interactions, such as end-of-life care planning protocols, appear to support greater SDM adoption success because they rely on workflows and processes that are already in practice. Adoption through incremental change, such as an evolutionary approach rather than a revolution, may be more likely to gain momentum over time [85].

### Implications for Future Research

Our review found that only a few randomized clinical studies were noted. Future research studies should assess the effect of SDM in multisite pragmatic RCTs.

This review identified a wide range of approaches to how SDM interventions are organized and delivered within the clinical setting. Future research could explore contextually aware tiered levels of intervention. This is important to standardize SDM care processes within specialties in order to support large-scale adoption of validated SDM tools and methods while addressing practical considerations within the constraints of each specialty.

Our review found that 17% (3/18) of the studies attempted to engage patients before a clinical encounter; however, no studies attempted to engage patients in decision-making after a clinical encounter [76,79,84]. Protracted decisions that include a series of small decisions align well with the use of patient portals to begin preparing patients for the SDM process. Another area for future research could be to understand the efficacy of postclinical encounter engagement to address the ongoing maintenance and reinforcement of decisions that have been made in collaboration with the clinician.

We found limited studies that included the identification and measurement of patient values. Thus, patient involvement in establishing their values and alignment of care with those values appear to be areas for additional focus in future research. Involving patients in goal setting is a key element to support patient engagement, which is essential to help patients manage their decisions outside of the clinician encounter [92].

### Strengths and Limitations of Approach

#### Strengths

The review followed rigorous standard scoping review methods set by the JBI, Arksey and O’Malley [59], and others [60,66,93,94]. We carried out a systematic search of multiple databases developed by a medical librarian and independent article screening and data extraction by at least 2 reviewers, with disagreement resolved through consensus. Our research team included investigators from an academic medical setting with extensive research experience in biomedical informatics, cognitive psychology, human factors, and health care.

#### Limitations

This scoping review may have missed relevant studies that are not indexed by the databases searched. Furthermore, the search retrieved studies published between January 1, 2009, and January 11, 2021; therefore, studies published outside this time frame were not included in our analysis. We did not search the gray literature or unpublished data sources. SDM studies that lacked an SDM tool, EHR implementation, and an actual clinical setting were not included. Implementation details were highly variable in terms of the approach and the level of detail described in the studies. SDM definitions and interpretations were variable, and we found limited references to validated SDM definitions, such as commonly cited definitions by the US National Institutes of Health or other credible sources.

### Conclusions

This scoping review revealed a wide range of approaches to the integration and implementation of SDM tools within an EHR workflow. We found that SDM tool integration methods ranged from simple scan and save methods to fully integrated and operable technical integration with an EHR system. Moreover, implementation approaches were highly variable, ranging from sending an email to clinicians to create awareness to more complete programs involving demonstrations, training, incentives, clinician-led promotion, and feedback sessions. Areas for future research include more RCTs, the development of contextually aware SDM interventions, longitudinal patient engagement with pre- and postvisit interventions, the study of patient-driven goals and values, and the subsequent impact on patient satisfaction levels. Future studies should detail pragmatic and actionable integration and implementation steps to advance the uptake of SDM.

## Appendix

Multimedia Appendix 1 Detailed search terms.

Multimedia Appendix 2 PRISMA-ScR (Preferred Reporting Items for Systematic Reviews and Meta-Analyses Extension for Scoping Reviews) checklist.

## Abbreviations

EHR: electronic health record
JBI: Joanna Briggs Institute
PRISMA-S: Preferred Reporting Items for Systematic Reviews and Meta-Analyses Search Extension
PRISMA-ScR: Preferred Reporting Items for Systematic Reviews and Meta-Analyses Extension for Scoping Reviews
RCT: randomized controlled trial
SDM: shared decision-making

## Data Availability

The datasets generated and analyzed during this study are available from the corresponding author on reasonable request.
