# Supplementary material for: Shared Decision-Making Tools Implemented in the Electronic Health Record: Scoping Review
Source: J Med Internet Res. 2025 Feb 21;27:e59956. doi: 10.2196/59956 (PMC11890150; doi:10.2196/59956)
Supplement: Multimedia Appendix 1 [file jmir_v27i1e59956_app1.docx]

**Appendix A**

Search strategies for **Shared Decision-Making Tools Implemented in the Electronic Health Record: A Scoping Review**

Sources: Medline (Ovid), Embase (Embase.com), CINAL Complete (Ebscohost), APA PsycINFO (Ebscohost), Scopus (scopus.com), Web of Science Core Collection (Clarivate).

Date limits: January 1, 2009 – January 11, 2021

All search ran on January 11, 2021.

**Ovid MEDLINE(R)** January 11, 2021

1 decision making, shared/ (678)

2 (shared adj4 (decision-making or problem-solving)).ti,ab,kw. (9175)

3 (shared adj3 decision*).ti,ab,kw. (9604)

4 ("patient decision* aid*" or (patient* adj2 "decision* aid*")).ti,ab,kw. (1002)

5 or/1-4 [Shared decision set 1] (10604)

6 Decision Support Systems, Clinical/ (8231)

7 Decision Support Systems, Management/ or management information systems/ or hospital information systems/ (15252)

8 decision support techniques/ or data interpretation, statistical/ (77986)

9 Decision Making, Organizational/ (11159)

10 (CDS or CDSS).ti,ab. or ((clinical or hospital) adj4 ("decision support system?" or "information system?")).ti,ab,kf,kw. (22908)

11 (decision-make* or (decision adj2 (maker* or making or support))).ti,ab,kw. (167345)

12 decision*.ti,ab. (393530)

13 "clinical informatics".ti,ab,kw. (539)

14 or/6-13 [Clin decision set] (498145)

15 "Decision Making, Computer-Assisted"/ (2798)

16 (((computerized or computer-assisted) adj2 decision*) or "computerized decision aid*").ti,ab,kw. (1068)

17 or/15-16 [Computerized decision set ] (3727)

18 Patient Participation/ (26518)

19 (patient* adj2 (activation or choice or empowerment or engagement or involvement or participat* or portal* or preference* or report* or shared)).ti,ab,kw. (219275)

20 (patient-portal* or patient-report* or parent-reported or patient-related or patient-choice*).ti,ab,kw. (54945)

21 patient-centered care/ or narrative medicine/ or patient navigation/ (20965)

Annotation: Exploded Mesh term for patient-centered care

22 (patient-center* or patient-focus* or medical-home*).ti,ab,kw. (22856)

23 or/18-22 [Patient participation set] (283896)

24 Electronic Health Records/ (20899)

25 medical record linkage/ or medical records systems, computerized/ or health information exchange/ (23537)

26 ((computer* or electronic or linkage) adj2 (health or medical) adj3 record?).ti,ab,kf. (38090)

27 "Meaningful Use"/ or (meaningful adj1 "use?").ti,ab,kf. (1905)

28 or/24-27 [EHR set] (66624)

29 medical records/ or medical records, problem-oriented/ (66667)

30 information systems/ or big data/ or community networks/ or geographic information systems/ or health information systems/ or knowledge bases/ or biological ontologies/ or gene ontology/ or medical informatics computing/ or public health informatics/ or hospital information systems/ or point-of-care systems/ (71403)

31 database management systems/ or data systems/ (7796)

32 Automation/ (18543)

33 informatics/ or medical informatics/ or health information exchange/ or medical informatics applications/ or medical informatics computing/ or nursing informatics/ (18395)

34 29 and (or/30-33) (2285)

35 ((computer* or electronic or linkage or automated) adj2 (clinical or health or medical or patient*) adj2 record?).ti,ab,kw. (39620)

36 or/34-35 [ EMR set ] (41761)

37 or/28,36 [EHR/EMR combined set] (70381)

38 ("health information technology" or "computerized patient").ti,ab. (3490)

39 ("30784423" or "28986341" or "27623534" or "25755233" or "25224366" or "24073039").ui. [exemplars] (6)

40 and/5,37 [SDM + EHR/EMR set] (224)

41 5 and (or/17,38) [SDM + computerized patient/health information technology set ] (86)

42 and/14,23,37 [Patient participation + Clin Decision + EHR set] (880)

43 and/14,23,38 [Patient participation + Clin Decision + computerized patient/health information technology set] (119)

44 and/17,23 [Patient participation +Computerized decision set] (178)

45 or/40-44 [near final set] (1221)

46 limit 45 to yr="2009 - 2020" [FInal set] (942)

47 or/39,46 [Final set includes exemplars] (942)

48 remove duplicates from 47 (933)

49 limit 48 to yr="2020 - 2021" (122)

Field codes: Medline(Ovid)

/ = Medical Subject Heading/MeSH | .ti,ab,kf,kw. = title, abstract, keyword, keywords assigned by indexers or authors

**Embase (embase.com)** January 11, 2021

#35 #33 NOT 'conference abstract'/it **34**

#34 #33 AND 'conference abstract'/it 12

#33 #30 AND [11-11-2020]/sd NOT [12-1-2021]/sd **46**

#32 #30 NOT 'conference abstract'/it **837**

#31 #30 AND 'Conference Abstract'/it **360**

#30 (#27 OR #28) AND [2009-2020]/py 1197

#29 #27 OR #28 1271

#28 #14 AND #20 AND #26 1006

#27 #5 AND #26 467

#26 #21 OR #22 OR #23 OR #24 OR #25 101398

#25 'meaningful use criteria'/de OR ((meaningful NEAR/1 use):ti,ab,kw) 2219

#24 ((computer* OR electronic OR linkage OR automated) NEAR/2 (clinical OR health OR medical OR patient*) NEAR/2 record*):ti,ab,kw 75500

#23 'electronic health record*':ti,ab,de,kw OR 'electronic medical record*':ti,ab,de,kw OR 'electronic patient record*':ti,ab,de,kw 95091

#22 ((computer* OR electronic OR linkage OR automated) NEAR/2 (clinical OR health OR medical OR patient*) NEAR/2 record*):ti,ab,kw 75500

#21 'electronic health record'/de OR 'electronic health record certification'/de 20094

#20 #15 OR #16 OR #17 OR #18 OR #19 426842

#19 'patient center*':ti,ab,kw OR 'patient focus*':ti,ab,kw OR 'medical home*':ti,ab,kw 31841

#18 'patient decision making'/de 10442

#17 'patient portal*':ti,ab,kw OR 'patient report*':ti,ab,kw OR 'parent reported':ti,ab,kw OR 'patient related':ti,ab,kw OR 'patient choice*':ti,ab,kw 91986

#16 (patient* NEAR/2 (activation OR choice OR empowerment OR engagement OR involvement OR participat* OR portal* OR preference* OR report* OR shared)):ti,ab,kw 357203

#15 'patient participation'/de 28341

#14 #8 OR #9 OR #10 OR #11 OR #12 OR #13 380229

#13 'clinical informatics':ti,ab,kw 664

#12 decision*:ti 84907

#11 ('decision making' NEAR/3 (clinical* OR medical* OR patient*)):ti,ab,kw 49553

#10 (decision* NEAR/2 (support OR aid? OR making OR modeling OR tool*)):ti,ab,kw 237592

#9 ((clinical OR medical) NEAR/2 decision*):ti,ab,kw 62329

#8 'clinical decision making'/de OR 'medical decision making'/de OR 'patient decision making'/de OR 'ethical decision making'/de OR 'family decision making'/de 145810

#7 cds:ti,ab OR cdss:ti,ab OR (((clinical OR hospital) NEAR/4 ('decision support system?' OR 'information system?')):ti,ab,de) 21334

#6 'clinical decision support system'/de OR 'decision support system'/de 26316

#5 #1 OR #2 OR #3 OR #4 17807

#4 'patient decision* aid*':ti,ab,kw OR ((patient* NEAR/2 'decision* aid*'):ti,ab,kw) 1304

#3 (shared NEAR/3 decision*):ti,ab,kw 14528

#2 (shared NEAR/4 ('decision making' OR 'problem solving')):ti,ab,kw 13755

#1 'shared decision making'/de 8009

Field codes: Embase (Elsevier)

de = subject headings | ti = title | ab = abstract | kw= keyword

**Cumulative Index of Nursing and Allied Health Literature (CINAHL) Complete (Ebscohost)** January 11, 2021

S29 S28 Limiters - Published Date: 20090101-20210131 259

S28 S26 OR S27 277

S27 S16 AND S20 AND S25 148

S26 S5 AND S25 155

S25 S21 OR S22 OR S23 OR S24 40,808

S24 (MH "Meaningful Use") OR TI "Meaningful Use" OR AB "Meaningful Use" 2,075

S23 (MH "Meaningful Use") 1,269

S22 TI ( ((computer* or electronic or linkage) N2 (health or medical) N3 record?) OR "electronic health record*" OR "electronic medical record*" OR "electronic patient record*" ) OR AB ( ((computer* or electronic or linkage) N2 (health or medical) N3 record?) OR "electronic health record*" OR "electronic medical record*" OR "electronic patient record*" ) 23,054

S21 (MH "Electronic Health Records") 25,912

S20 S17 OR S18 OR S19 132,368

S19 TI ( (patient-portal* or patient-report* or parent-reported or patient-related or patient-choice*) (patient-center* or patient-focus* or medical-home*) ) OR AB ( (patient-portal* or patient-report* or parent-reported or patient-related or patient-choice*) (patient-center* or patient-focus* or medical-home*) ) 89

S18 TI ( (patient* N2 (activation or choice or empowerment or engagement or involvement or participat* or portal* or preference* or report* or shared)) ) OR AB ( (patient* N2 (activation or choice or empowerment or engagement or involvement or participat* or portal* or preference* or report* or shared)) ) 115,534

S17 (MH "Consumer Participation") 20,737

S16 S6 OR S7 OR S8 OR S9 OR S10 OR S11 OR S12 OR S13 OR S14 OR S15 63,522

S15 TI (decision*) 37,692

S14 TI ( (((computerized or computer-assisted) N2 decision*) or "computerized decision aid*") ) OR AB ( (((computerized or computer-assisted) N2 decision*) or "computerized decision aid*") ) 559

S13 TI "clinical informatics" OR AB "clinical informatics" 237

S12 ( ((clinical or hospital) N4 ("decision support system?" or "information system?")) ) OR ( ((clinical or hospital) N4 ("decision support system?" or "information system?")) ) 1,754

S11 TI ( CDS OR CDSS OR ((clinical or hospital) N4 ("decision support system?" or "information system?")) ) OR AB ( CDS OR CDSS OR ((clinical or hospital) N4 ("decision support system?" or "information system?")) ) 3,469

S10 (MH "Decision Support Techniques") 6,921

S9 (MH "Decision Making, Organizational") 3,496

S8 (MH "Decision Making, Computer Assisted") 1,332

S7 (MH "Management Information Systems") OR (MH "Clinical Information Systems") OR (MH "Health Information Systems") 11,880

S6 (MH "Decision Support Systems, Clinical") OR (MH "Decision Support Systems, Management")

5,862

S5 S1 OR S2 OR S3 OR S4

7,558

S4 TI ( ("patient decision* aid*" or (patient* N2 "decision* aid*")) ) OR AB ( ("patient decision* aid*" or (patient* N2 "decision* aid*")) ) 711

S3 TI (shared N3 decision*) OR AB (shared N3 decision*) 5,981

S2 TI ( (shared N4 (decision-making or problem-solving)) ) OR AB ( (shared N4 (decision-making or problem-solving)) ) 5,666

S1 (MH "Decision Making, Shared") 1,896

Field codes: CINAHL Complete (Ebscohost)

MH = MeSH heading | SU = | TI = Title | AB = Abstract

**APA PsycINFO (Ebscohost)** January 11, 2021

S26 S24 OR S25 Limiters - Published Date: 20090101-20210131 94

S25 S15 AND S19 AND S23 79

S24 S7 AND S23 42

S23 S20 OR S21 OR S22 4,770

S22 ( TI "Meaningful Use" OR AB "Meaningful Use" ) OR KW "Meaningful Use" 266

S21 ( I ( ((computer* or electronic or linkage) N2 (health or medical) N3 record?) OR "electronic health record*" OR "electronic medical record*" OR "electronic patient record*" ) OR AB ( ((computer* or electronic or linkage) N2 (health or medical) N3 record?) OR "electronic health record*" OR "electronic medical record*" OR "electronic patient record*" ) ) OR KW ( ((computer* or electronic or linkage) N2 (health or medical) N3 record?) OR "electronic health record*" OR "electronic medical record*" OR "electronic patient record*" ) ) 4,288

S20 (DE "Electronic Health Records") 1,583

S19 S16 OR S17 OR S18 332,672

S18 ( TI ( (patient-portal* or patient-report* or parent-reported or patient-related or patient-choice*) (patient-center* or patient-focus* or medical-home*) ) OR AB ( (patient-portal* or patient-report* or parent-reported or patient-related or patient-choice*) (patient-center* or patient-focus* or medical-home*) ) ) OR ( (patient-portal* or patient-report* or parent-reported or patient-related or patient-choice*) (patient-center* or patient-focus* or medical-home*) ) 33

S17 ( TI ( ((patient* or client*) N2 (activation or choice or empowerment or engagement or involvement or participat* or portal* or preference* or report* or shared)) ) OR AB ( ((patient* or client*) N2 (activation or choice or empowerment or engagement or involvement or participat* or portal* or preference* or report* or shared)) ) ) OR ( ((patient* or client*) N2 (activation or choice or empowerment or engagement or involvement or participat* or portal* or preference* or report* or shared)) ) 332,669

S16 DE "Client Participation" 2,364

S15 S8 OR S9 OR S10 OR S11 OR S12 OR S13 OR S14 48,508

S14 TI decision* 44,851

S13 ( TI ( (((computerized or computer-assisted) N2 decision*) or "computerized decision aid*") ) OR AB ( (((computerized or computer-assisted) N2 decision*) or "computerized decision aid*") ) ) OR KW ( (((computerized or computer-assisted) N2 decision*) or "computerized decision aid*") ) ) 276

S12 ( TI "clinical informatics" OR AB "clinical informatics" ) OR KW "clinical informatics" 31

S11 ( ( ((clinical or hospital) N4 ("decision support system?" or "information system?")) ) OR ( ((clinical or hospital) N4 ("decision support system?" or "information system?")) ) ) OR KW ( ((clinical or hospital) N4 ("decision support system?" or "information system?")) ) 294

S10 ( TI ( CDS OR CDSS OR ((clinical or hospital) N4 ("decision support system?" or "information system?")) ) OR AB ( CDS OR CDSS OR ((clinical or hospital) N4 ("decision support system?" or "information system?")) ) ) OR KW ( CDS OR CDSS OR ((clinical or hospital) N4 ("decision support system?" or "information system?")) ) 1,648

S9 (DE "Information Systems") AND (TI ( decision N2 (making OR support) ) OR AB ( decision N2 (making OR support) ) OR KW ( decision N2 (making OR support) )) 574

S8 (DE "Decision Support Systems") 3,364

S7 (S1 OR S2 OR S3 OR S4 OR S5 OR S6) 4,190

S6 ( TI ( ("patient decision* aid*" or (patient* N2 "decision* aid*")) ) OR AB ( ("patient decision* aid*" or (patient* N2 "decision* aid*")) ) ) OR KW ( ("patient decision* aid*" or (patient* N2 "decision* aid*")) ) OR ( TI ( ("client decision* aid*" or (client* N2 "decision* aid*")) ) OR AB ( ("client decision* aid*" or (client* N2 "decision* aid*")) ) ) OR KW ( ("client decision* aid*" or (client* N2 "decision* aid*")) ) 331

S5 ( TI (shared N3 decision*) OR AB (shared N3 decision*) ) OR KW (shared N3 decision*) 3,375

S4 ( TI ( (shared N4 (decision-making or problem-solving)) ) OR AB ( (shared N4 (decision-making or problem-solving)) ) ) OR KW ( (shared N4 (decision-making or problem-solving)) ) 3,293

S3 DE "Patient Centered Care" 259

S2 DE "Group Decision Making" AND (TI ( shared OR patient* ) OR AB ( shared OR patient* ) OR KW ( shared OR patient* )) 311

S1 DE "Decision Making" AND (TI decision* N2 shared OR AB decision* N2 shared OR KW decision* N2 shared ) 2,076

Field codes: APA PsycINFO (Ebscohost)

DE = Descriptor | SU = Subject | TI = title | AB = abstract | KW = keyword

**Scopus (scopus.com)** January 11, 2021

603 document results

( ( TITLE-ABS-KEY ( ( shared W/4 ( decision-making OR problem-solving ) ) OR ( shared W/3 decision* ) OR ( "patient decision* aid*" OR ( patient* W/2 "decision* aid*" ) ) ) ) AND ( ( TITLE-ABS-KEY ( ( ( ( computerized OR computer-assisted ) W/2 decision* ) OR "computerized decision aid*" ) ) ) OR ( TITLE-ABS-KEY ( ( ( computer* OR electronic OR linkage OR automated ) W/2 ( clinical OR health OR medical OR patient* ) W/2 record* ) ) ) ) ) OR ( ( TITLE-ABS-KEY ( ( shared W/4 ( decision-making OR problem-solving ) ) OR ( shared W/3 decision* ) OR ( "patient decision* aid*" OR ( patient* W/2 "decision* aid*" ) ) ) ) AND ( TITLE-ABS-KEY ( ( ( health OR medical ) W/3 record* ) OR "meaningful use" ) ) ) OR ( ( TITLE-ABS-KEY ( ( patient* W/2 ( activation OR choice OR empowerment OR engagement OR involvement OR participat* OR portal* OR preference* OR report* OR shared ) ) OR ( patient-portal* OR patient-report* OR parent-reported OR patient-related OR patient-choice* ) OR ( patient-center* OR patient-focus* OR medical-home* ) ) ) AND ( TITLE-ABS-KEY ( ( ( ( computerized OR computer-assisted ) W/2 decision* ) OR "computerized decision aid*" ) ) ) AND ( TITLE-ABS-KEY ( ( ( health OR medical ) W/3 record* ) OR "meaningful use" ) ) ) AND PUBYEAR > 2008

Field codes: Scopus (Elsevier)

TITLE-ABS-KEY = title, abstract, keyword

**Web of Science Core Collection: Citation Indexes (Clarivate)** January 11, 2021

Science Citation Index Expanded (SCI-EXPANDED) --1900-present

Social Sciences Citation Index (SSCI) --1900-present

Arts & Humanities Citation Index (A&HCI) --1975-present

Emerging Sources Citation Index (ESCI) --2015-present

# 6 **257** #5 Indexes=SCI-EXPANDED, SSCI, A&HCI, ESCI Timespan=**2009-2021**

# 5 282 #4 OR #1

# 4 226 #2 AND #3

# 3 37,779 TS=( ( ( "health record*" OR "medical record*" ) AND ( computerized OR computer-assisted OR electronic* OR automated ) ) OR "meaningful use" )

# 2 14,736 TS=( ( shared NEAR/3 ( decision-making OR problem-solving ) ) OR ( shared NEAR/3 decision* ) OR ( "patient decision* aid*" OR ( patient* NEAR/2 "decision* aid*" ) ))

# 1 272 TOPIC: (( ( shared NEAR/4 ( decision-making OR problem-solving ) ) OR ( shared NEAR/3 decision* ) OR ( "patient decision* aid*" OR ( patient* NEAR/2 "decision* aid*" ) ) ) AND ( ( ( ( computerized OR computer-assisted ) NEAR/2 decision* ) OR "computerized decision aid*" ) OR ( ( ( computer* OR electronic OR linkage OR automated ) NEAR/2 ( clinical OR health OR medical OR patient* ) NEAR/2 record* ) ) ))

Field codes: Web of Science Core Collection (Clarivate)

TS = Topic search
